# Minimally-verbal children with autism show deficits in theta and gamma oscillations during processing of semantically-related visual information

**DOI:** 10.1038/s41598-019-41511-8

**Published:** 2019-03-25

**Authors:** Silvia Ortiz-Mantilla, Chiara Cantiani, Valerie L. Shafer, April A. Benasich

**Affiliations:** 1 Center for Molecular & Behavioral Neuroscience, Rutgers University-Newark, Newark, NJ USA; 2 Scientific Institute, IRCCS E. Medea, Child Psychopatology Unit, Bosisio Parini, Lecco, Italy; 3 The Graduate Center, City University of New York, New York, USA

## Abstract

To acquire language, children must build phonemic representations of their native language, learn to associate auditory words to visual objects and assemble a lexicon. It is not clear however, whether the limited linguistic ability seen in minimally-verbal (MV) children with Autism Spectrum Disorder (ASD) relates to deficits in cortical representation of an object and/or in linking an object to its semantic information. This EEG-based study investigated neural mechanisms underlying visual processing of common objects in MV-ASD and control children. Ten MV-ASD children, 4- to 7- years-old and 15 age/gender-matched controls, were presented with a picture-word matching paradigm. Time-frequency analyses were conducted at the sources generating the event-related responses at both early and late visual processing. Permutation testing identified spectral power and phase coherence clusters that significantly differed between the groups. As compared to controls, MV-ASD children exhibited smaller amplitudes and longer source latencies; decreased gamma and theta power with less theta phase coherence in occipital regions, and reduced frontal gamma power. Our results confirm that visual processing is altered in MV-ASD children and suggest that some of the linguistic differences observed in these children arise from impaired object/label cortical representations and reduced allocation of attention, which would impact lexical acquisition.

## Introduction

Most children follow a predictable path as they acquire language, a path largely constrained by brain maturation and environmental experience^1^. Nevertheless, a subset of children with developmental disorders, including those with Autism Spectrum Disorders (ASD) falter in achieving language milestones. ASD is a heterogeneous neurodevelopmental disorder characterized by behavioral, communication and social-interaction impairments (American Psychiatric Association, DMS-5, 2013). The majority of studies in children with ASD have been conducted with participants who have been able to develop relatively good language skills. However, minimally-verbal (MV) children with ASD, which easily represents 25% of children diagnosed with ASD, are infrequently studied^2^. As these children have difficulty following instructions, it is a great challenge to accurately ascertain how much incoming speech information they perceive, decode, and/or comprehend^3^.

To acquire language, typically developing children build phonemic maps of the sounds of their native language in auditory cortex. These maps facilitate rapid encoding of stimulus features, promoting fast automatic processing of speech^4^. In the first year of life, infants also begin to learn word-object pairings^5^, linking semantic features of objects, including the object’s name to auditory representations^6^. Thus, even before beginning to talk, infants understand and respond to many words^7^. However, whether the limited ability to express and perhaps comprehend language observed in MV children with ASD is related to absent or deficient cortical representations of object features, and/or to deficits in the ability to couple an object and its semantic information or other factors is not yet fully understood.

Examination of neural correlates underlying object processing can shed light on deficits in speech processing. Measures of neuronal oscillations provide a critical tool for assessing neural mechanisms that support early language acquisition^4^. Brain oscillations are periodic fluctuations in neural excitability, mainly a result of synchronized post-synaptic activity^8^. Oscillations in the theta range (4–8 Hz) are thought to be globally involved in long-range information transfer of synchronized neuronal activity across brain areas^8,9^. Gamma oscillations (>30 Hz) primarily reflect neuronal activity occurring at local level^10^ although they may also play a role in the coupling of remote cortical areas^11^. Whereas theta oscillations play, among other functions, a role in declarative and episodic memory processing^8,9^, gamma oscillations have been associated with cognitive and perceptual operations including attention^12^, perceptual binding^13^, object recognition^14^, comparison of memory content with stimulus-related information^15,16^, and encoding, retention and retrieval of information^10^. Studies in the visual domain have demonstrated higher levels of “early” gamma power to objects for which participants already have achieved long-term memory representations as compared to objects for which no memory representations are available^15^. Moreover, it has been suggested that reduced gamma power during “late” visual processing of a picture may reflect deficient activation of memory traces related to that picture, for example, retrieval of the picture name^13,16^.

As neural oscillations are known to be atypical in ASD^17^, in the current study, we examined the oscillatory underpinnings of the event-related (ERP) responses to visual images at both early and late stages of visual processing. Participants were shown pictures of common objects/animals prior to the presentation of semantically congruent or incongruent auditory words with the picture priming the following word. We hypothesized that enhanced gamma oscillatory activity would be observed to established long-term memory representations that link visual image features with other semantic information about the object/animal, including auditory labels. Consequently, if the limited linguistic ability of MV-ASD children is related to absent or deficient cortical representations of an object’s visual features, then reduced gamma activation would be expected during early stages of visual processing; in contrast, if a rich cortical representation of visual features was established but activation of memory traces related to the image’s semantic features were faulty or missing, we expect to see reduced gamma activation during later stages of processing.

## Methods

### Participants

Twenty five children between four and seven years of age participated in this study: 10 (7 boys) MV-ASD children (mean age: 6 years, 4 months; SD: 13 months) and 15 (9 boys) typically developing, age/gender-matched control (CTL) children (mean age: 5 years, 11 months; SD: 14 months). In the original study, 15 MV-ASD children were recruited, however, data from 4 MV-ASD participants could not be acquired and for 1 participant, data was sub-optimal. For that reason, in the MV-ASD group we acquired complete data for only 10 children. We retained all 20 children included in our previous report^18^, with the exception of one CTL child with a high level of noise in the gamma band, and added six children that had originally been tested as age/gender-matched CTLs to the present CTL sample. The groups did not differ in age (*F*_(1,24)_ = 0,588, *p* = 0.451).

Participants were born full-term into monolingual English families, had no history of head trauma or other neurological or co-morbid genetic conditions and lived in the metropolitan New York/New Jersey area. Children with ASD were recruited through the NJ Autism and Language Genetics Study (NJLAGS), the International Autism Network (IAN) and private schools for children with ASD. All MV-ASD participants were diagnosed with ASD by a developmental pediatrician, neurologist, or a licensed clinical psychologist prior to recruitment and were already enrolled in self-contained, special-education classes for children with ASD. Nine of 10 children with ASD had less than five functionally/intelligible words and one child had an expressive vocabulary of 20 words. Since standardized testing is extremely difficult for these children and results of adapted assessments are not reliable^19^, no measures of IQ could be provided for this group.

CTL children had typical development, and scored within the normal range for non-verbal IQ (Kaufman Brief Intelligence Test, K-BIT or Stanford-Binet Intelligence Scales) and language (Peabody Picture Vocabulary Test, Fourth Edition [PPVT-4]—Preschool Language Scale, Fourth Edition [PLS-4] or Clinical Evaluation of Language Fundamentals, Fourth Edition [CELF-4]). Written consent was obtained from all parents/caregivers before their child’s participation. Children in the CTL group also provided assent. All study procedures were approved by the Rutgers University and City University of New York (CUNY) Institutional Review Boards and were performed in accordance with the Declaration of Helsinki.

### Stimuli

**Figure 1:**
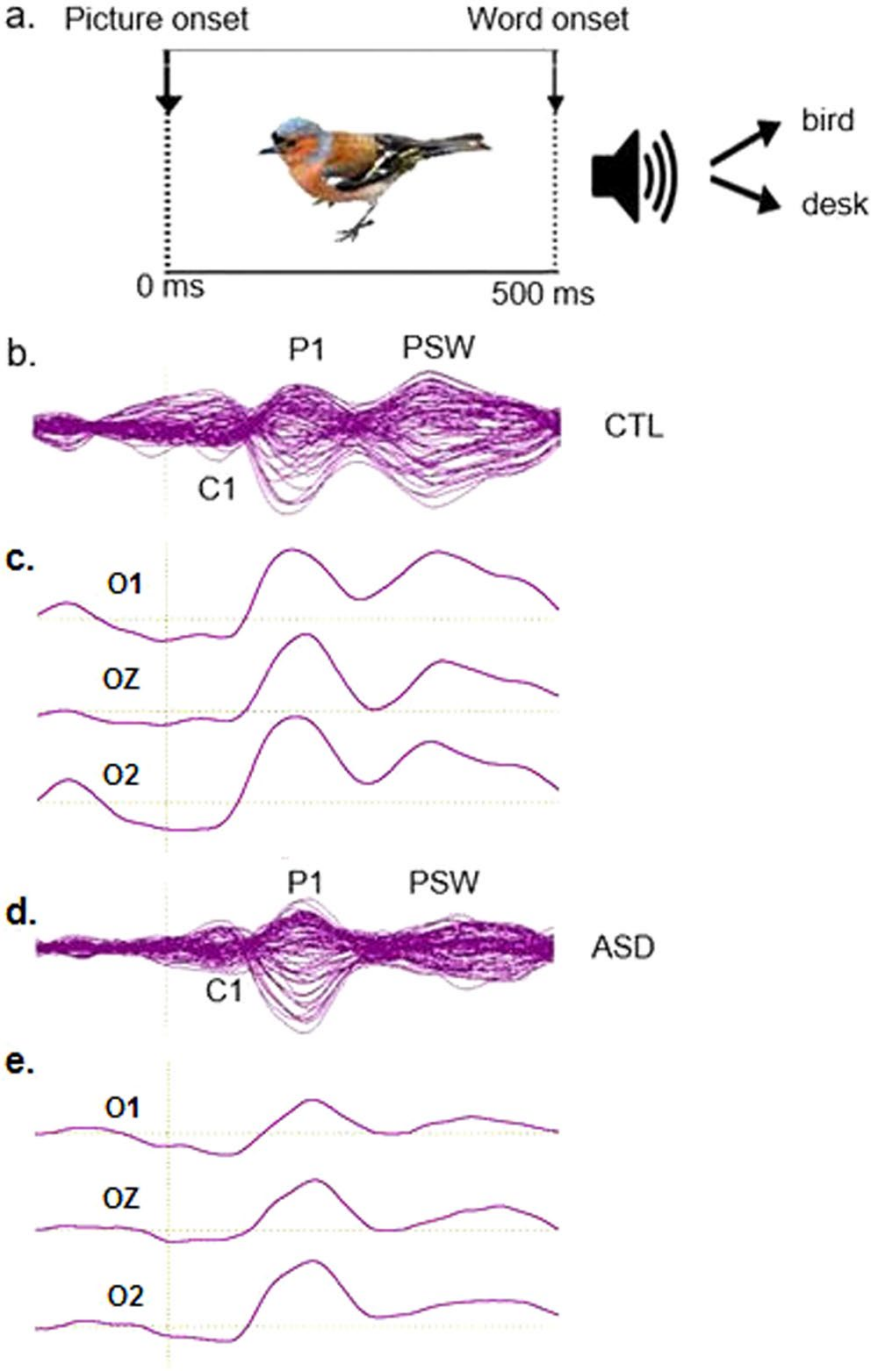
(**a**) Exemplary trial (modified from Cantiani, *et al*.^18^). Visual responses were examined in the first 500 ms after picture onset. (**b**) Butterfly plot showing for the control (CTL) group, overlaid (64 channels) grand average ERP waveforms, in response to visual stimuli. (**c**) Grand average ERP waveforms are shown at occipital channels (O1: left occipital, O2: right occipital, Oz: midline occipital) for the CTL group. Three main ERP components show a negative polarity for C1, and positive polarity for P1 and the positive slow wave (PSW). (**d**) Butterfly plot showing for the minimally verbal autism (MV-ASD) group, overlaid grand average ERP waveforms, including all 64 channels. (**e**) Grand average ERP waveforms shown at occipital channels (O1: left occipital, O2: right occipital, Oz: midline occipital) for the MV-ASD group. The early sensory responses (P1) were present in both groups but the late visual response (PSW) was clearly decreased in the MV-ASD group.

For the visual stimuli we used 60 full-color digitized photographs of animals or inanimate objects against a white background, presented on a 54 × 30 cm Samsung monitor placed 81 cm from the participant. The same picture was repeated twice, once followed by a congruent word and once followed by an incongruent word^18^. The pictures and words chosen were highly familiar and easily understood by two-year old children. The 120 pictures were arranged in four pseudo-randomized blocks, each containing 30 pictures and were delivered by E-prime software (version 1.1). At the beginning of each block, a fixation point (central cross) was presented for 500 ms. Then, pictures were centrally presented on the computer monitor for the entire duration of the trial with the intertrial constant at 2000 ms (total trial length). An auditory word that either matched or mismatched the picture began after 500 ms of the picture onset (Fig. 1a). Participants were asked to look at the pictures while the EEG was recorded, but no overt behavioral response was required.

### Event-related Potentials (ERP) recording and processing

Prior to the ERP session, the MV-ASD group underwent sensory desensitization training to facilitate net application and EEG/ERP acquisition^20^. The ERP session lasted 15 minutes and the entire session was video recorded to make sure that children were looking at the visual displays during the trials. When necessary, the researcher paused the experiment while the entertainer redirected the child attention to the visual display. Six practice trials were presented at the beginning of the experiment, to familiarize the participants with the paradigm. Data was recorded in an acoustically and electrically shielded chamber, with Net Station 3.0.2, using a 64 Ag/AgCl channel EGI sensor net (Electric Geodesic Inc.); the vertex electrode was used as the online reference, with a sampling rate of 250 Hz and online band-pass filtered (0.1–100 Hz). Impedances were kept below 50 kOhm.

After recording, EEG signals were processed to extract ERPs, using Brain Electrical Source Analysis (BESA) 5.2 software (see^18^). Eye blinks were removed from the raw EEG data by an automatic correction algorithm based on principal component analyisis (PCA) method. Data were band-pass filtered at 0.3–30 Hz and re-referenced offline to an average reference. No more than 30% of noisy channels were interpolated using the BESA spline interpolation method (mean of 16 channels interpolated: 19 for MV-ASD and 15 for CTL group). The continuous EEG was segmented into epochs with a baseline of 100 ms and 1500 ms post-stimulus time and time-locked to the picture onset. Epochs with signals exceeding 300 µV from the baseline were excluded.

### Source localization of ERP generators

Generators of the visual response were investigated by mapping ERP data onto a 4-shell ellipsoidal 6-year-old head template provided by BESA Research 6.1 software. The ERP data was band-pass filtered 1–15 Hz and peaks for the responses to the visual stimuli identified from the grand average file in the first 500 ms of the trial. The average number of trials for the CTL group were 95.6 trials (standard deviation: 7.1) and for the MV-ASD, 85.4 trials (standard deviation: 11.7). A time window of ±20 ms around the selected peak^21,22^ was used to allow the best signal-to-noise ratio (SNR) during discrete dipole fitting^23^. For the purposes of this paper, we focused our analysis exclusively on the responses to the visual stimuli. There were two main reasons for choosing this methodological approach: (1) The visual part of the experiment had better SNR than the visual-auditory segment as 120 trials containing pictures were shown to the children. During the following visual-word part of the experiment, 500 ms after picture onset, half of the words were a match to the pictures, and half were mismatched limiting the analyses to 60 trials per condition, which would decrease the SNR. Moreover, after data cleaning, the number of clean trials were further reduced. (2) Source localization of the ERP generators to the visual stimuli could be reliably modeled with a 3-dipole model that explained most of the variance for both groups. However, to explain the variance during the more complex auditory-visual processing, a dipole model (with 5 or more sources) would have been necessary and we did not feel that this approach was the best way to explain the current visual data.

### Time Frequency Analyses

To examine time-frequency changes during early (0–250 ms) and late (300–500 ms) visual processing the 64-channel recording was processed as follows: a fixed spatial filter (source montage) created from the averaged ERP data during dipole fitting was applied to each individual’s unfiltered recording to transform the ongoing EEG into a source space. This spatial filter separates activity from the different brain regions identified by the dipole model. A complex demodulation method with 1-Hz-wide frequency bins (2–90 Hz) and 50 ms time resolution (from −300 to 500 ms) was used to transform single trials into time-frequency representations^24^. A low cutoff of 1.0–1.5 Hz, was applied during scanning of the EEG in cases where prominent slow-wave activity was present^24,25^. Spectral power and phase stability dynamics were assessed by temporal spectral evolution (TSE) and inter-trial phase locking (ITPL). TSE was used to examine event-related changes in oscillatory amplitude (power) of the different frequency bands relative to the baseline^26^. The TSE value is comprised of both induced (non-phase locked) and evoked (phase locked) changes in oscillatory amplitude in response to a stimulus^13,26,27^. Inter-trial phase locking (ITPL), was used to determine inter-trial phase coherence. ITPL measures how consistently the phase at different frequency bands locks to stimulation presented across trials, thus representing the temporal relationship of oscillations of a given frequency^13,26^.

### Statistical analyses

Studies conducted in the ASD population has been reported using both balanced samples, in which age/gender matched participants are evenly matched across group or with unbalanced samples in which the number of participants differ between the ASD and CTL groups. To determine if statistical analyses were influenced by the number of participants in the CTL sample^28^, source localization and time frequency analyses were conducted with both balanced (10 MV-ASD and 10 age/gender matched CTL participants^18^) and unbalanced groups (10 MV-ASD and 15 CTL participants). Strength and latency of the sources were examined using one-way ANOVAs in SPSS Statistics 23 (IBM® SPSS® Corp) software with the alpha-level for *p* significance set at 0.05. Time-frequency regions with significant changes in spectral power and inter-trial phase coherence were detected via permutation testing and cluster analysis in BESA Statistics 2.0 (BESA®), which uses parameter-free permutation testing on the basis of Student’s *t* test^29^ to provide results corrected for multiple comparisons (http://www.besa.de/products/besa-statistics/brochures/). The *p* statistics values reported in the time-frequency domain were derived from the permutation testing.

## Results

We found that overall, results of the statistical analyses were analogous whether using the smaller balanced or the larger unbalanced control sample, with one key exception being the group significance of ITPL in the gamma band. Given that this ITPL difference for gamma may provide insights into emerging phase coherence in the gamma band over development, we decided to present the results for both the balanced and unbalanced groups.

### Localization of the ERP Generators and Source Analyses

In both groups, visual stimuli elicited an ERP response characterized by three main components shown in butterfly plots with all channels overlaid irrespective of their polarity (Fig. 1b for CTL; Fig. 1d for MV-ASD groups). At posterior channels (occipital left, occipital right and occipital midline), the polarity of the visual responses was clearly characterized by a small negative deflection (C1) at ~80 ms followed by a bifurcated positive deflection in which the first component was a sharp positivity (P1) that peaked at ~180 ms, and the second, a positive slow wave (PSW) peaking at ~400 ms (Fig. 1c for CTL; Fig. 1e for MV-ASD groups). Results of the ERP analysis were previously reported^18^).

**Figure 2:**
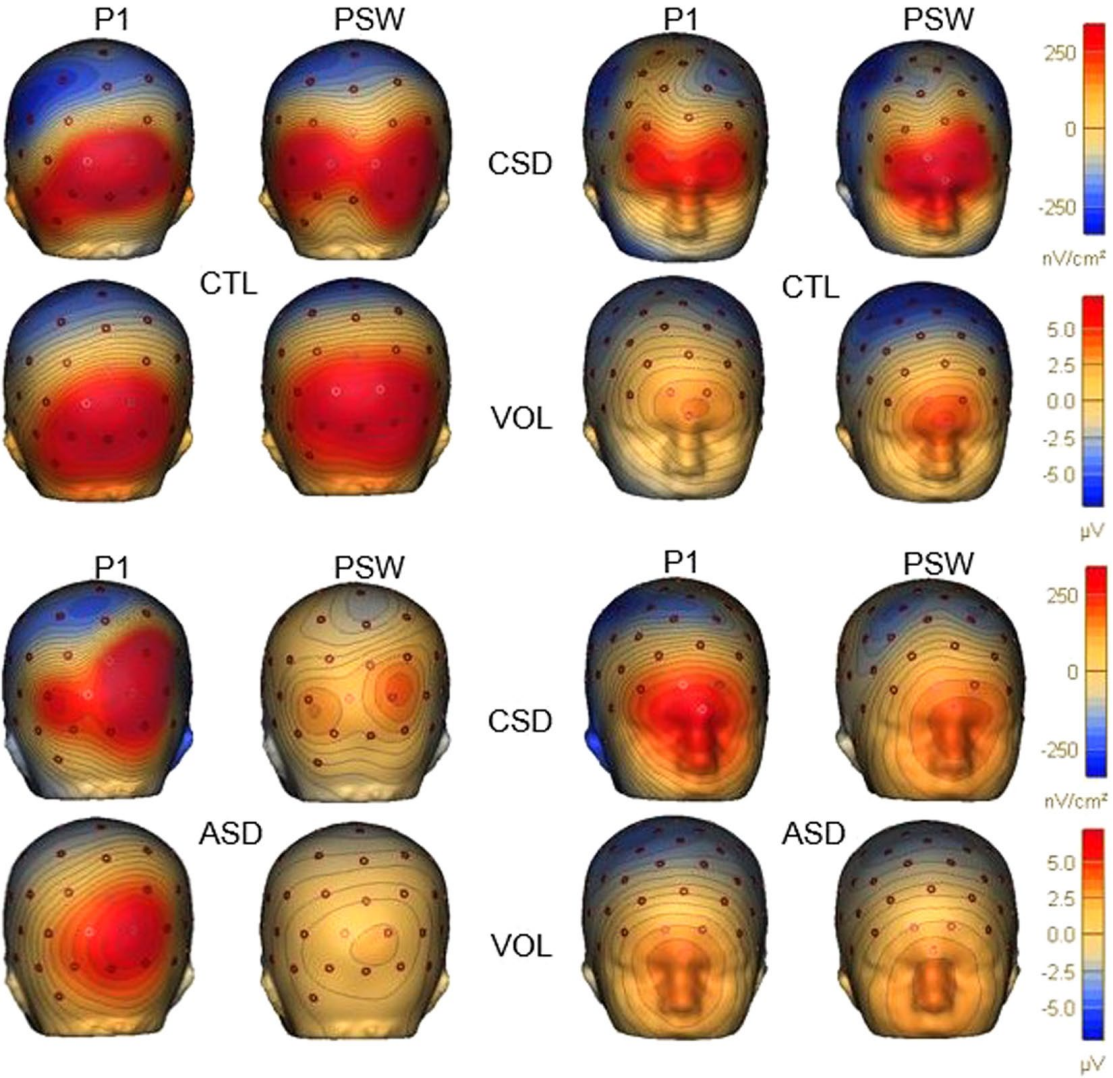
Voltage (VOL) and current source density (CSD) maps: VOL and CSD maps showing the scalp surface and Laplacian voltage distribution at the P1 and PSW peaks of the occipital cortex (2 left columns) and frontal (2 right columns) at the level of anterior cingulate cortex (ACC). VOL and CSD maps for P1 (left) and PWS (right) are shown above for CTL and below for MV-ASD groups plotted on a reconstructed head model. Magnitude of the electrical response is given in microvoltios (µV) in the VOL map and in nanovoltios/cm^2^ (nV/cm^2^) in the CSD map with red shades indicating more and blue shades less electrical activity.

The scalp voltage (VOL) and current source density (CSD) maps for P1 and PSW peaks displayed a distinctive topographical distribution for each group. In the VOL map, CTLs showed increased posterior activity close to the midline for both P1 and PSW peaks, while in the CSD map, a midline topography was observed for P1 and a more bilateral distribution for PSW. The posterior topography suggests that neural activity originated in occipital cortex. The MV-ASD group displayed midline activity in the VOL map for both peaks, although less prominently than the CTLs particularly for PSW, but lateralized topography was evident in the CSD map for both the P1 and PSW peaks (Fig. 2).

**Figure 3:**
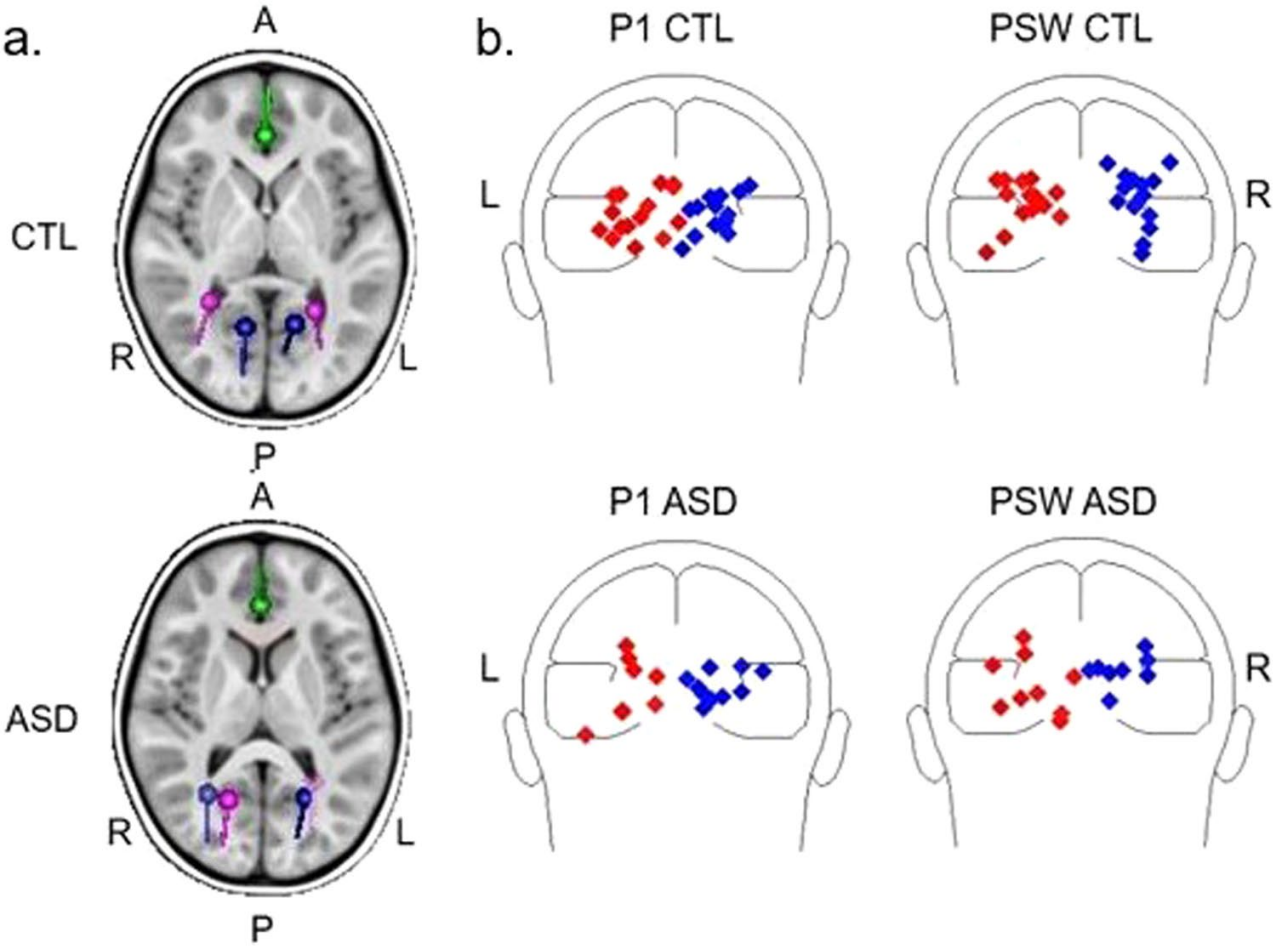
(**a**) Dipole model in the grand average ERP waveform: Discrete dipole solutions for the occipital P1 (blue) and PSW (magenta) at the peak amplitude based on the grand average for CTL (above) and MV-ASD (below) localized in a 6-year-old MRI template. The ACC dipole is shown in green. Sources of the occipital P1 are more medially located for the CTL group (above) whereas sources for the PSW are more laterally placed. This pattern was not precisely defined in the MV-ASD group (below). A: anterior; P: posterior; L: left; R: right. (**b**) Individual source localization of the P1 and PSW visual responses: Individual locations for the CTL group are shown superimposed on a schematic head above and for the MV-ASD group are shown below; dipoles in red are located at left (L) and in blue at right (R) occipital cortices. Larger variability in dipole locations was observed in the MV-ASD than in the CTL group for both positive responses but in particular for the PSW.

**Figure 4:**
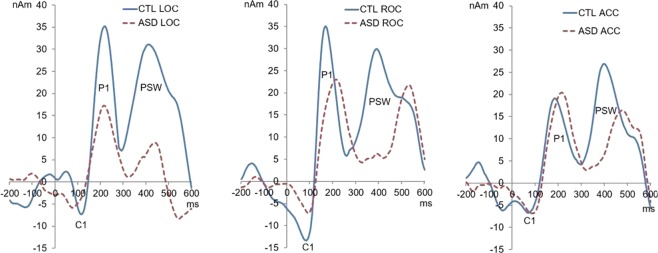
Source waveforms of the visual response: source waveforms at left (LOC) and right (ROC) occipital cortices and frontally at the level of anterior cingulate cortex (ACC) are shown as a solid blue line for the CTL group and a dotted red line for the MV-ASD group. Positivity is plotted up; amplitude of the source dipole moment is given in nanoamperes (nAm) in the *y-axis* and latency in milliseconds (ms) in the *x-axis*.

Sources of the visual responses were initially identified for each group in the grand-averaged ERP file. A 3-dipole model (Fig. 3a), with 2 dipoles located at left (LOC) and right (ROC) occipital cortices, and a mid-frontal dipole placed at the level of anterior cingulate cortex (ACC), explained most of the variance for each peak (residual variance for C1: CTL: 4.49%; MV-ASD: 8.01%; P1: CTL: 5.39%, MV-ASD: 5.51%; PSW: CTL: 3.78%, MV-ASD: 6.69%). The morphology of the source waveforms for both groups closely followed the ERP waveforms indicating a good model fit for the data (Fig. 4).

Subsequently, dipoles were fitted at the individual level. The occipital dipoles were fitted freely but the frontal dipole was fixed based on the grand average solution of the CTL group because free fitting of this source was not stable at the individual level. Due to small amplitude, the C1 response could be reliably modeled on only a few children (CTL:8/15; MV-ASD: 5/10) thus, statistical analyses were not conducted for the C1 component. The overlaid individual dipoles for CTLs clustered similarly to the topography observed in the CSD maps: dipoles were more medially located for P1 but more laterally placed for the PSW while the MV-ASD group showed a more dispersed distribution (Fig. 3b).

### Comparison between MV-ASD and CTL using balanced groups

#### Source analysis

**Figure 5:**
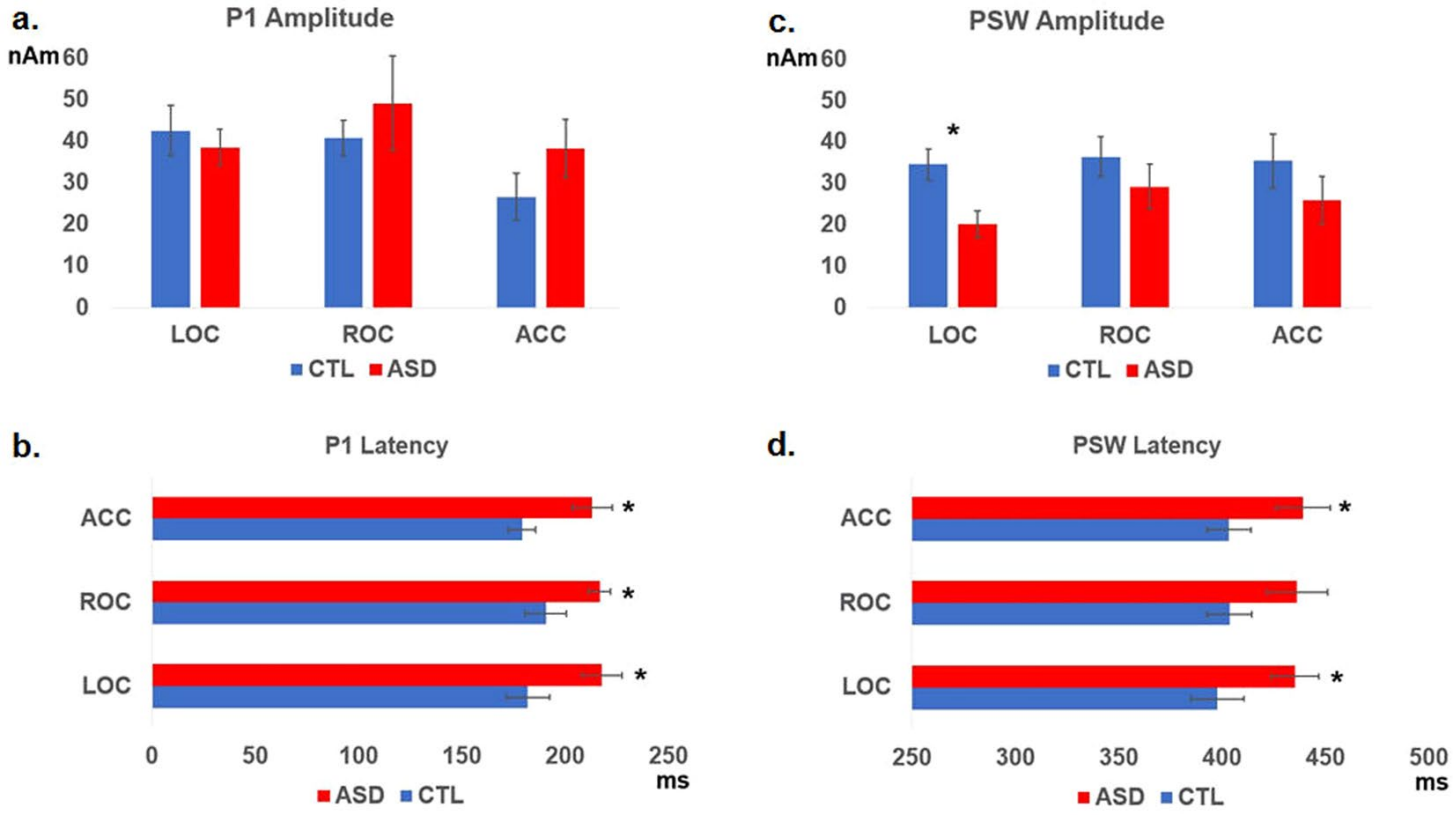
Group differences in amplitude and latency of source responses: (**a**) Bar graphs comparing P1 source amplitude between MV-ASD and CTL groups. No group difference was found in left (LOC), right (ROC) or anterior cingulate cortex (ACC) sources. Blue bars represent CTL group and red bars MV-ASD group. Amplitude is given in nanoamperes (nAm) in the y-axis. (**b**) Comparison of source P1 latency between MV-ASD and CTL groups. The MV-ASD group (red) showed longer latencies in LOC, ROC and ACC sources than the CTL group (blue). Significance is marked with a black asterix. Time is given in miliseconds (ms) in the x-axis. (**c**) Bar Graph showing comparison of PSW source amplitude between MV-ASD and CTL groups. CTL group (blue) had larger amplitude in left (LOC) occipital source than MV-ASD (red). Amplitude is given in nanoamperes (nAm) in the y-axis. (**d**) Comparison of source PSW latency between MV-ASD and CTL groups. The MV-ASD group (red) showed longer latencies than the CTL group (blue) in LOC and ACC sources Significance is marked with a black asterisk. Time is given in miliseconds (ms) in the x-axis. Standard error bars are represented by a black line in each bar graph.

We found that P1 latency and PSW latency and amplitude differed between the groups, but no group difference in P1 amplitude was seen (Fig. 5a). As compared to CTLs, the MV-ASD group showed later P1 latency (Fig. 5b) at LOC (*F*_(1,19)_ = 6.12, *p* = 0.024), ROC (*F*_(1,19)_ = 5.14, *p* = 0.036), and ACC (*F*_(1,19)_ = 8.49, *p* = 0.009). For the PSW peak, the MV-ASD group had smaller amplitude (*F*_(1,19)_ = 8.73, *p* = 0.008) at LOC (Fig. 5c) and longer latency at LOC and ACC (*F*_(1,19)_ = 4.56, *p* = 0.047; *F*_(1,19)_ = 4.44, *p* = 0.049) respectively, than the CTL group (Fig. 5d). Latency and amplitude means and standard deviation for P1 and PSW peaks in each group are presented in Table 1.

**Table 1:** Comparison of amplitude and latency of source positive peaks (P1, PSW) between balanced MV-ASD and CTL groups.

		Amplitude (nAm) (sd)			Latency (ms) (sd)		
		CTL	MV-ASD	*p*	CTL	MV-ASD	*p*
P1	LOC	42.59 (19.37)	38.58 (27.49)	0.711	182.80 (33.15)	218.00 (30.42)	0.024*
	ROC	40.80 (13.69)	49.18 (35.79)	0.498	190.80 (32.00)	216.80 (17.05)	0.036*
	ACC	26.67 (17.90)	38.31 (22.07)	*0.211*	179.20 (20.81)	213.20 (30.47)	0.009*
PSW	LOC	34.63 (11.71)	20.10 (10.22)	0.008*	398.00 (40.53)	435.60 (36.44)	0.047*
	ROC	36.46 (15.40)	29.21 (17.00)	0.330	403.60 (34.01)	436.40 (46.59)	0.089
	ACC	35.51 (20.55)	25.92 (18.51)	0.287	403.20 (33.67)	439.20 (42.24)	0.049*

nAm: nanoamperes; ms: milliseconds; sd: standard deviation; CTL: control group; MV-ASD: minimally verbal autism spectrum disorder group; P1: first positive peak; PSW: positive slow wave; LOC: left occipital cortex; ROC: right occipital cortex; ACC: anterior cingulate cortex; *significant difference between the groups, shown in Italic text.

### Time-Frequency Analysis

The MV-ASD group showed less phase coherence (ITPL) than CTLs in the delta-theta (2–8 Hz, 0–500 ms) range during both early (0–250 ms) and late (300–500 ms) visual processing in LOC (*p* = 0.001), ROC (*p* = 0.003), and ACC, (*p* = 0.028). No significant group differences in phase coherence were found in the gamma range.

In a similar fashion, reduced spectral power was seen for MV-ASD children compared to CTLs in the delta-theta range in LOC (*p* = 0.023) during early visual processing (2–6 Hz; 0–250 ms) and in ROC (*p* = 0.026), during late (2–6 Hz; 300–500 ms) visual processing. In the gamma band, in both early and late processing time frames (30–90 Hz; 100–500 ms), the MV-ASD group also showed less spectral power than the CTL group in LOC (*p* = 0.013), ROC (*p* = 0.023) and ACC (*p* = 0.036).

### Comparison between MV-ASD and CTL using unbalanced groups

#### Source analysis

Corresponding to the results observed for the balanced groups, the latency and strength of the P1 and PSW peaks differed between the unbalanced groups. As compared to CTLs, the MV-ASD group showed later P1 latency at LOC (*F*_(1,24)_ = 5.25, *p* = 0.031) and ROC (*F*_(1,24)_ = 6.82, *p* = 0.016), smaller PSW amplitude (*F*_(1,24)_ = 7.39, *p* = 0.012) and longer PSW latency (*F*_(1,24)_ = 5.28, *p* = 0.031) at LOC. For the ACC latency, the difference observed approached significance with the MV-ASD group tending to have later peak latencies (P1: *p* = 0.054; PSW: *p* = 0.057) than CTLs (Table 2).

**Table 2:** Comparison of amplitude and latency of source positive peaks (P1, PSW) between unbalanced MV-ASD and CTL groups.

		Amplitude (nAm) (sd)			Latency (ms) (sd)		
		CTL	MV-ASD	*p*	CTL	MV-ASD	*p*
P1	LOC	44.29 (23.07)	38.58 (27.49)	0.580	187.73 (33.52)	218.00 (30.42)	0.031*
	ROC	43.47 (19.59)	49.18 (35.79)	0.611	185.07 (35.60)	216.80 (17.05)	0.016*
	ACC	28.94 (22.31)	38.31 (22.07)	0.312	188.53 (29.31)	213.20 (30.47)	0.054
PSW	LOC	33.97 (13.76)	20.10 (10.22)	0.012*	402.67 (34.23)	435.60 (36.44)	0.031*
	ROC	35.36 (13.36)	29.21 (17.00)	0.322	406.67 (39.15)	436.40 (46.59)	0.098
	ACC	32.69 (18.51)	25.92 (18.51)	0.379	409.33 (32.38)	439.20 (42.24)	0.057

nAm: nanoamperes; ms: milliseconds; sd: standard deviation; CTL: control group; MV-ASD: minimally verbal autism spectrum disorder group; P1: first positive peak; PSW: positive slow wave; LOC: left occipital cortex; ROC: right occipital cortex; ACC: anterior cingulate cortex; *significant difference between the groups, shown in Italic text.

### Time-frequency analysis

**Figure 6:**
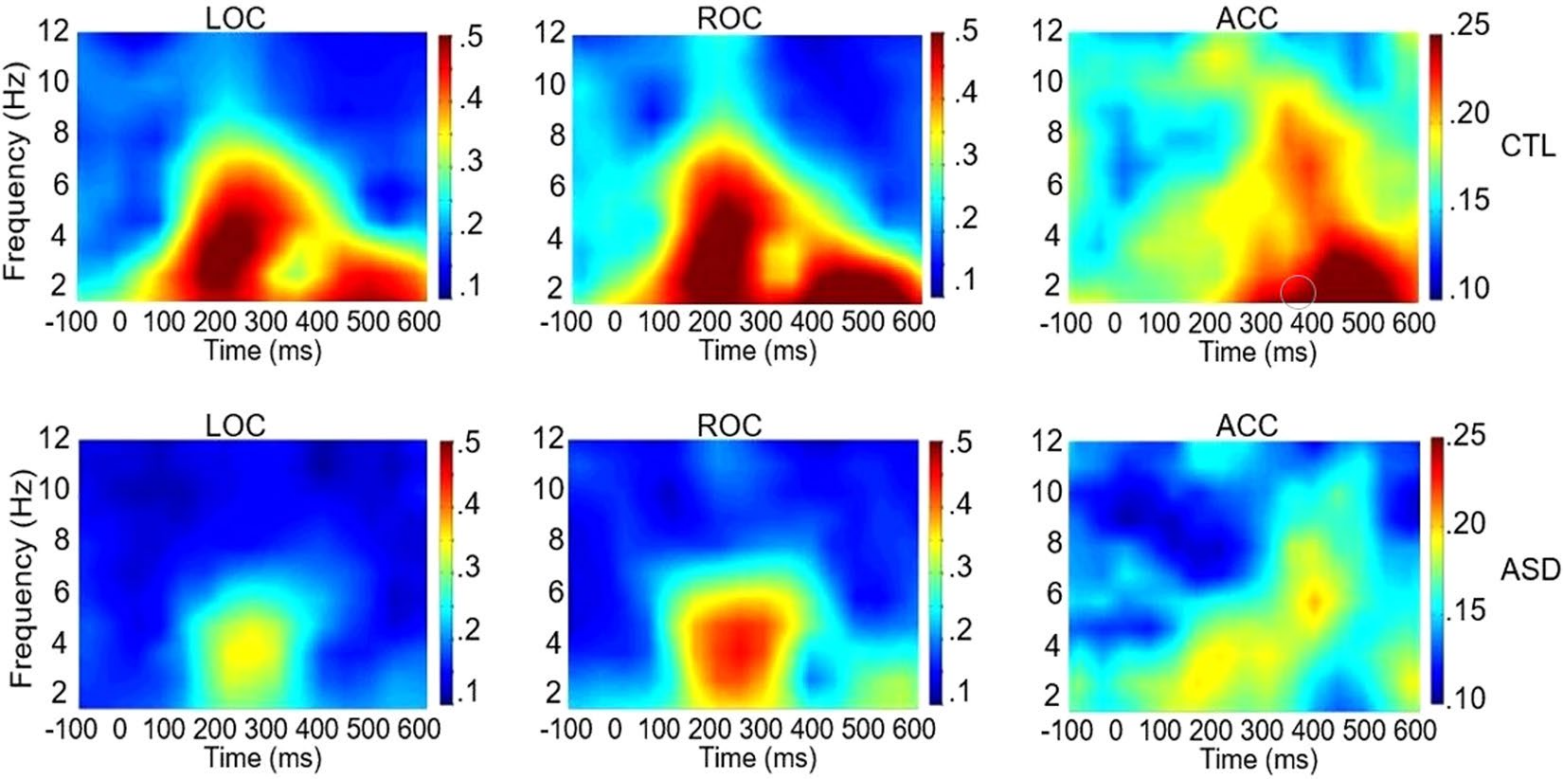
Inter-trial phase coherence (ITPL) in theta range: Time-frequency plots across participants showing ITPL in theta frequency band at left (LOC) and right (ROC) occipital cortices and frontal at the level of anterior cingulate cortex (ACC) during picture processing. Plots for the control (CTL) group are shown in the first row and for the MV-ASD group in the second row. The color scale represents ITPL values with red shades indicating more phase coherence and blue shades indicating less phase coherence. Time is given in milliseconds (ms) in the *x-axis* and frequency is given in Hz in the *y-axis*.

Analysis in the time frequency domain conducted with unbalanced groups echoed the results found for the balanced groups in all but one of the analyses. The MV-ASD group showed less phase coherence than CTLs in the delta-theta range (0–500 ms, 2–8 Hz) during early and late visual processing (Fig. 6) in LOC (*p* = 0.000), ROC (*p* = 0.001), and ACC (*p* = 0.023). However, in the gamma range, MV-ASD children showed significantly less ITPL than CTLs (*p* = 0.001) in LOC at the early stages of visual processing (0–200 ms; 39–75 Hz), a result that was not evident in the balanced group analysis.

**Figure 7:**
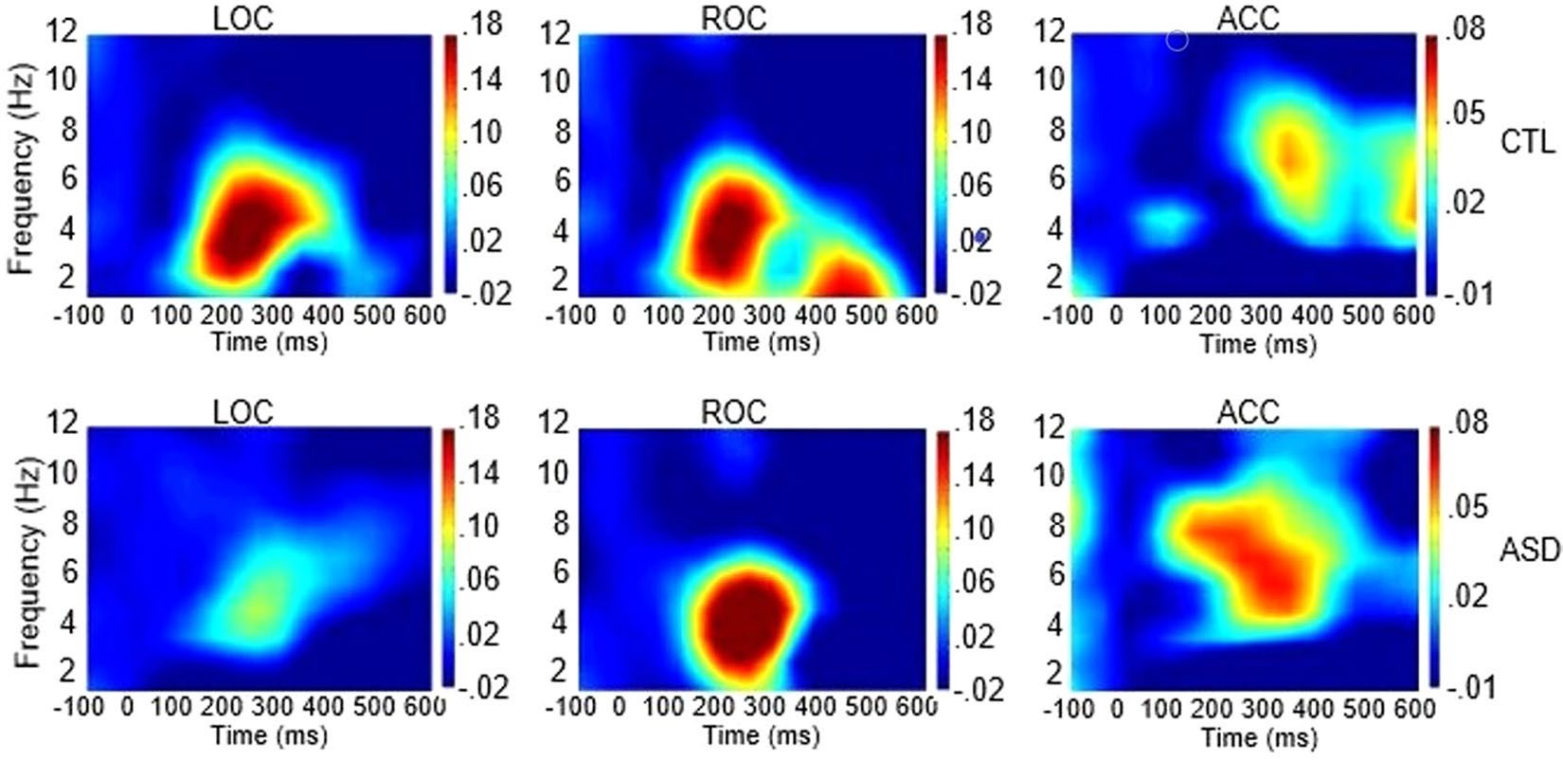
Temporal Spectral Evolution (TSE) in theta range: Time-frequency plots across participants showing spectral power in theta frequency band at left (LOC) and right (ROC) occipital cortices and frontal at the level of anterior cingulate cortex (ACC) during picture processing. Plots for the control (CTL) group are shown in the first row and for the MV-ASD group in the second row. The color scale represents percentage of amplitude change as compared to the baseline with warm colors indicating more power and blue colors indicating less change in spectral power. Time is given in milliseconds (ms) in the *x-axis* and frequency is given in Hz in the *y-axis*.

**Figure 8:**
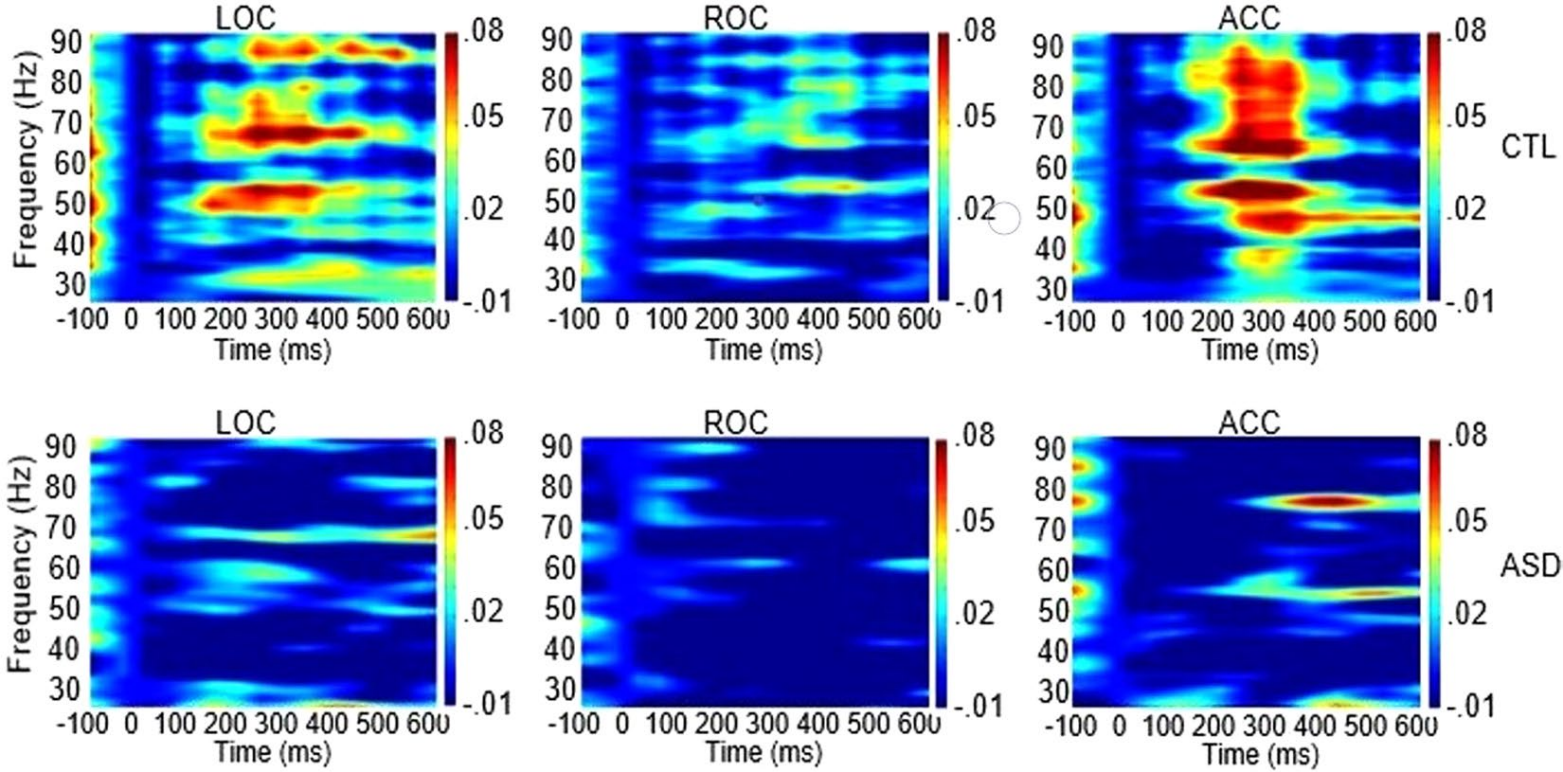
Temporal Spectral Evolution (TSE) in gamma range: Time-frequency plots across participants showing spectral power in the gamma frequency band at left (LOC) and right (ROC) occipital cortices and frontally at the level of anterior cingulate cortex (ACC) during picture processing. Plots for the control (CTL) group are shown in the first row and for the MV-ASD group in the second row. The color scale represents percentage of amplitude change as compared to the baseline with red shades indicating more power and blue shades indicating less changes in spectral power. Time is given in milliseconds (ms) in the *x-axis* and frequency is given in Hz in the *y-axis*.

Less spectral power was seen for MV-ASD children than for CTLs in the delta-theta range (2–6 Hz; 0–250 ms) in LOC (*p* = 0.018) during early, and in ROC (*p* = 0.006) during late (2–6 Hz; 300–500 ms) visual processing (Fig. 7). In the gamma band (30–90 Hz; 100–500 ms), the MV-ASD group also recruited significantly less power than the CTL group (Fig. 8) in LOC (*p* = 0.001), ROC (*p* = 0.001) and ACC (*p* = 0.000).

## Discussion

Children with ASD who have limited linguistic abilities are often characterized as “low functioning” primarily because their level of performance cannot be determined using standard cognitive and language assessments^19^. It is unknown whether or how MV-ASD children perceive and comprehend incoming speech information^30^ and importantly, which neural mechanisms related to language processing are most impacted. This study begins to address these questions by examining event-related oscillatory dynamics to pictures in a picture-word priming paradigm. The major findings revealed lower spectral power in theta and gamma bands and reduced theta phase coherence in occipital cortical areas during both early and late visual processing for the MV-ASD group as compared to CTLs. MV children with ASD showed limited visual processing in time frames associated with cortical representation of objects^15^ as well as linking/retrieval of semantic-related information^13,16^. The MV-ASD group also showed reduced gamma power in the ACC suggesting that fewer attentional resources were devoted to the processing of semantically-related visual information. A decrease in attention allocation could impact both the initial cortical mapping process as well as later retrieval of less densely coded object representations.

Disruptions in oscillatory synchrony have been proposed as a key neural correlate underlying difficulties in sensory and perceptual information processing in ASD^31–33^. Oculo-motor control, fixation patterns, and visuo-spatial cortical mapping are also altered in autism^34^. Our results suggest that MV children with ASD experience alterations during both early and late stages of visual processing that may include difficulty encoding stimulus features and mapping objects to related semantic information. However, it is still unclear to what extent these patterns may be causally related to their difficulties in language learning and expression.

Event-related changes in evoked (phase locked) and induced (non-phase locked) oscillatory amplitude examined with temporal spectral evolution (TSE), are thought to reflect the extent and magnitude of the neural ensembles involved in stimulus processing^13,26,27^. Encoding of the physical characteristics of an object occurs primarily during the earlier stages of visual processing, mostly supported by increases in time-locked, evoked oscillatory activity^13,16^. We found that compared to CTLs, MV-ASD children had longer P1 latencies suggesting a delay in encoding visual information that may be modulated by attention^35,36^. MV-ASD children also demonstrated less spectral power than CTLs in the early evoked (phase locked) gamma response, which has been related to the processing of image features^37^ and to the binding process required for building object representations^16^, which subsequently are stored in memory^13^. Objects for which adult participants have already achieved long-term memory representations (i.e. objects whose features have been cortically mapped and thus, when presented, match memory content) elicit larger early (<150 ms) evoked gamma responses over occipital cortex than objects for which no memory representation is available^15^. These results suggest that feedback from memory systems may enhance the gamma response seen in visual areas^15,16^. Similarly, in the auditory domain, increased early high-gamma power may indicate cortical mapping of familiar native phonemic features^4,38^. The finding of reduced evoked gamma oscillations during early visual processing in MV-ASD children suggests that a temporally synchronized, but smaller neuronal ensemble, was engaged in the sensory evaluation of the stimulus which may reflect compromised encoding of stimulus features and/or establishment of its cortical representation.

Neurophysiological indices of induced, non-phase locked oscillatory activity during late visual processing have also been related to object representation. Increases in induced gamma activity from 200–400 ms after sensory input have been proposed as the underlying mechanism for feature binding during generation of object representation^13^. Reduction of power in the late gamma response may also reflect inadequate activation of object-related memory traces, including retrieval of the object name^16^, which can be influenced by top-down mechanisms of attentional selection, in particular, when expectations are present^39^. In our paradigm, a word was expected to follow the picture, and thus, a particular word would be “primed” if the pictured object was already in the child’s mental lexicon. When designing the paradigm, we carefully selected picture-word pairs that were highly familiar and easily understood by typically developing 2-year-olds^18^. If MV-ASD children have faulty encoding of object features and/or inadequate, poorly articulated memory representations of familiar objects, it would not be surprising if information related to the object is not quickly retrieved. We propose, that the low verbal skills that characterized this cohort of children may be due, in part, to deficiencies in object-feature encoding and in the establishment of object memory representations expected to be highly familiar to young children. Alternatively, even if object representations are stored in memory, these children may have diminished accessibility to these representations or may lack the ability to retrieve semantic-related object information. Future studies will be needed to clarify these hypotheses.

The concomitant enhancement of gamma power in the ACC seen in CTLs supports the premise that this is an attention-modulated process. As compared to CTLs, MV-ASD children showed reduced gamma power in the ACC, a structure that has been implicated in allocation and control of attentional resources^40,41^. During visual processing, both occipital and frontal gamma activation has been reported^14^. Atenttional modulation of the occipital response, by increasing power to the attended stimuli^13,42^, may originate in frontal cortex^16^ supporting the role of frontal areas in internal object representation^14^. Atypical orientation of attention has also been found in ASD^36,43,44^. Our finding of reduced gamma power in the ACC in MV-ASD children implicate weaker top-down mechanisms of attentional control and/or less allocation of attention to the visual stimuli.

We found that for most of our analyses, results were similar when using the smaller (balanced) or larger (unbalanced) control sample. However, the significant group difference in phase stability of early gamma oscillations across trials (ITPL) revealed when analysis were conducted with unbalanced samples was not seen using the balanced sample. This may imply that the difference observed was unreliable or alternatively, it may be that the use of a larger CTL sample provided more power^28^ and facilitated the visualization of emerging phase coherence in the gamma band. ITPL, as a measure of phase stability, represents the temporal relationship of oscillations of a given frequency^45^. Very few studies have investigated ITPL in gamma during early visual processing in children. For instance, a study examining lifespan differences in visual processing, found less ITPL in gamma in children (mean age: 11.7 years) than in young adults (mean age: 23.4 years), demonstrating that phase stability was not fully mature at that age^45^. The fact that less phase synchrony in gamma was seen in children than adults may reflect age-related differences in myelination that would affect the generation of synchronized neuronal activity^46–48^. Since participants in our study were even younger (mean age: 5 years and 11 months) than those included in the Werkle-Bergner *et al*.^45^ study, it would not be surprising that phase stability in the gamma range was still under development and therefore, more clearly seen when analyses were conducted with a larger sample.

Limitations of this study include the small sample size of the MV-ASD group. EEG/ERP data were collected as part of a feasibility study aiming to explore techniques/paradigms to investigate the extent of linguistic abilities in MV-ASD children. Assembling and testing even this small cohort was quite challenging, given the difficulty in communication and the prevalent sensory issues in this clinical population. Simultaneous eye tracking while recording the EEG could had been helpful, but sensory issues ubiquitous in ASD children limit the feasibility of adding another device to the head. The entire session was video-recorded and manually examined to check that children were looking at the visual displays during trial onset. Moreover, the visual stimulus remained in view on the monitor for the duration of the trial. It has been established that when a stimulus is constantly presented at central locations a relatively short amount of time is sufficient to obtain reliable evoked responses; even so, it is posible that differences in eye positions between the groups could have influenced the visual responses^34^. An additional limitation is that our sample of ASD children included only those with minimal language. The majority of ASD studies have been conducted in high-functioning verbal populations thus facilitating access to neurotypical controls that can easily be age-matched to the ASD participants’ verbal and cognitive levels. In our ASD sample, 9 of the children had expressive language of less than 5 words (the 10^th^ child had about 20 words), and significant behavioral problems. For this reason, assessing IQ/cognitive performance would have been not only extremely challenging but mostly inaccurate. Even when standardized testing is feasible using an adapted format, the results are often not reliable^19^. We matched groups for both age and gender, but we did not consider it appropriate to match them on maturational or language levels because that would have required, based on their very minimal expressive language abilities, using infants or toddlers as controls. Moreover, given the lack of reliable standardized testing, we did not have information on language comprehension. In fact, that was one of the significant aims of this research study—to find a way to adequately assess language comprehension in children who were essentially non-verbal. Therefore, it is as yet unknown whether the mechanisms underlying picture-related semantic processing seen in this group are similar to those used by ASD children with better language abilities. Despite these limitations, our in-depth oscillatory analyses align with reports in the literature, and nicely complement and extend the findings from our previously reported sensor-level averaged ERP data^18^.

## Conclusions

Our results confirm that visual processing is altered in MV-ASD children and suggest that at least some of the linguistic differences observed in this group might arise from impaired object-label representations in the cortex accompanied by reduced attentional specificity and control. This study also provides a basis for designing future studies in which neural indices of information processing can be directly measured in a broader sample of children that includes verbal and non-verbal ASD groups across a larger age range. These findings advance our understanding of the neural mechanisms that support sensory/perceptual processing of semantically-related visual information in MV-ASD children as well as in age-matched control children who show typical language acquisition. Detecting the earliest signs of deviations from the normative timeline of developmental milestones is critical for implementing methods to correct and/or ameliorate atypical trajectories that will improve long-term developmental outcomes.

## Data Availability

Data files are securely stored per IRB guidelines at the Infancy Studies Lab at Rutgers University Rutgers-Newark. Access will be granted upon request to the senior author.
